# Synergistic Effects of Selected Nonthermal Technologies Combined with Soursop Leaf Extract on the Quality and Shelf Life of Refrigerated Pacific White Shrimp

**DOI:** 10.3390/foods14081388

**Published:** 2025-04-17

**Authors:** Abubakar Saleh Ahmad, Thanasak Sae-Leaw, Yadong Zhao, Lukai Ma, Bin Zhang, Hui Hong, Soottawat Benjakul

**Affiliations:** 1 International Center of Excellence in Seafood Science and Innovation (ICE-SSI), Faculty of Agro-Industry, Prince of Songkla University, Hat Yai, Songkhla 90110, Thailand; ahmadabubakarsale@gmail.com (A.S.A.); thanasak.s@psu.ac.th (T.S.-L.); 2 Key Laboratory of Health Risk Factors for Seafood of Zhejiang Province, College of Food Science and Pharmacy, Zhejiang Ocean University, Zhoushan 316022, China; yadong@kth.se (Y.Z.); zhangbin@zjou.edu.cn (B.Z.); 3 Key Laboratory of Green Processing and Intelligent Manufacturing of Lignan Specialty Food of Ministry and Rural Affairs, College of Light Industry and Food, Zhongkai University of Agriculture and Engineering, Guangzhou 510225, China; m1991lk@163.com; 4 Beijing Laboratory of Food Quality and Safety, College of Food Science and Nutritional Engineering, China Agricultural University, Beijing 100083, China; hhong@cau.edu.cn; 5 Department of Food and Nutrition, Kyung Hee University, Seoul 02447, Republic of Korea

**Keywords:** Pacific white shrimp, nonthermal treatments, soursop leaf extract, melanosis, quality, shelf life

## Abstract

The effectiveness of multi-targeted treatments including pulsed electric field (PEF), soursop leaf extract (SLE), vacuum impregnation (VI), and modified atmosphere packaging (MAP), with and without cold plasma (CP) treatment, on the quality and shelf life of Pacific white shrimp (*Penaeus vannamei*) during refrigerated storage for 21 days was investigated. PEF inhibited melanosis and reduced the initial bacterial load, as evidenced by lower melanosis scores and total bacterial counts in the treated samples. Integrating 1% SLE through VI effectively lowered color alteration, retarded melanosis, and preserved textural integrity in the SLE-treated samples (*p* < 0.05). SLE1 (1%) significantly reduced lipid oxidation, as witnessed by lower thiobarbituric acid reactive substances (*p* < 0.05) and minimal fatty acid profile changes. MAP3, comprising CO_2_/N_2_/Ar (60%/30%/10%), combined with CP treatment, ensured microbiological quality and maintained total viable count within the acceptable limit (6 Log CFU/g) throughout the storage time of 21 days. Notably, the PEF-SLE1-VI-MAP3-CP sample exhibited superior quality preservation, as shown by a lower pH and total volatile base content than the others. Sensory evaluation confirmed that the PEF-SLE1-VI-MAP3-CP sample remained sensorially acceptable during storage. Thus, this multi-hurdle approach demonstrated the synergistic potential of integrating nonthermal processing technologies with plant extracts, contributing to the extended shelf life and safety of the refrigerated shrimp for up to 21 days.

## 1. Introduction

Pacific white shrimp (PWS, *Penaeus vannamei*) is a popular commercial shrimp species accounting for 80% of global shrimp production [1]. It is widely consumed due to its appealing taste and flavor. However, the shelf life of the shrimp is limited; this limit is associated with various factors involving melanosis, microbiological spoilage, lipid oxidation, and enzymatic activity [2,3]. Polyphenoloxidase (PPO), which is concentrated under the carapace of the shrimp cephalothorax, causes melanosis, characterized by ‘black spots’ on the shrimp’s surface [4]. Melanosis results in significant economic losses to shrimp farmers and traders [5]. Microbial contamination is another important factor detrimental to shrimp quality and safety. Several categories of food spoilage bacteria, especially the psychrotrophic bacteria such as *Pseudomonas* spp. [3], *Shewanella putrefaciens* [6], *Aeromonas* [7], Enterobacteriaceae [3], lactic acid bacteria (LAB), and *C. perfringens* [8] have been identified in PWS, raising concerns regarding the microbiological quality and safety of shrimp for consumption. Key spoilage indicators in shrimp include volatile basic nitrogenous compounds, indole, and biogenic amines, which are triggered by microbial contamination [9]. Postmortem changes mediated by endogenous enzymes in shrimp can alter the tissue structure and compromise the shrimp’s texture, resulting in the loss of firmness as well as sensorial acceptability. Various methods have been employed to address these challenges and preserve shrimp quality during extended storage. These involve the use of cold storage combined with chemical or natural preservatives. Sodium metabisulfite is one of the most effective and commonly used chemical preservatives to prevent melanosis and quality loss in shrimp [10]. The adverse effects associated with sulfites, particularly to allergic individuals, have limited their use. Natural preservatives like essential oils, chitosan, chitooligosaccharides, bioactive peptides, plant extracts, and polyphenols having antioxidant, antimicrobial, and PPO inhibitory activities could retard quality deterioration in shrimp and extend their shelf life [11].

Soursop (*Annona muricata* L.) leaf extract (SLE) exhibits potential bioactivity due to its abundant phytochemicals such as tannins, glucosinolates, phenolic compounds, polysterols, acetogenins, and alkaloids [12]. SLE (1%) has been documented to inhibit melanosis and elongated the storage of PWS for 12 days, while the control sample could be kept for 9 days when stored at 4 ᵒC [13]. In general, the efficacy of plant extracts declined as the storage period increased, leading to the increased proliferation of microorganisms, melanosis, and other quality losses [14]. Innovative nonthermal technologies could be used together with the plant extract to enhance the effectiveness of the SLE for inhibiting melanosis and extending the shelf life of PWS. Nonthermal treatments, including pulsed electric field (PEF) [14], modified atmosphere packaging (MAP) [15,16], and cold plasma (CP) [17], showed their efficacy in shrimp quality maintenance and nutrient retention when integrated with plant extracts.

PEF entails the use of brief bursts of a high-voltage electric field to create micropores on the shrimp’s shell through an electroporation mechanism [1,18], facilitating the absorption of the active compounds of the plant extract into the shrimp muscle. Additionally, electroporation can cause the inactivation of bacterial cells if the electric field strength and the pulsed duration are sufficient to induce permeabilization of the cell membrane to allow the penetration of the antibacterial agent and destabilize the cells’ metabolic function [19,20]. In general, to enhance the penetration of target compounds into shrimp or other seafoods, vacuum impregnation (VI) has been employed. When the vacuum is applied, the air in the pores is removed. The solution containing the preservative or other target compounds can suddenly pass through the pore generated by PEF after the vacuum is released [21]. Thus, PEF, along with VI treatment, could enhance the penetration of the bioactive compounds into the shrimp shell and muscle, thereby improving the efficacy of the SLE.

MAP plays an important role in seafoods’ preservation. It involves sealing the product in a packaging system and altering the ambient gas surrounding the product. The main gases used include O_2_, CO_2_, and N_2_. MAP rich in CO_2_ and deficient in O_2_ can lower the physiological and biochemical changes in the food product [22], inhibit the growth of aerobic bacteria [22], retard lipid oxidation, and mitigate melanosis in shrimp [23]. N_2_ can be used to substitute for O_2_ to prevent oxidation and growth of aerophiles [24]. Inert gases such as argon (Ar), helium (He), neon (Ne), and xenon (Xe) have been increasingly applied in MAP [25]. Ar is widely used because of its chemical inertness with food [26], and it displaces oxygen, thereby helping to maintain the food quality.

Cold plasma (CP) is an emerging technology considered to be the fourth state of matter alongside traditional solid, liquid, and gas, that is capable of generating reactive oxygen species (ROS) and reactive nitrogen species (RNS) [25]. The potential of dielectric barrier CP for microbial decontamination [16] was documented. CP could inactivate microorganisms by forming lesions in the cell membrane and increasing the oxidation of cellular lipids and nucleic acids [26]. CP can be used to sanitize packaging material and decontaminate the food surface through the generation of active species including charged particles, free radicals, photons, chemical species, and UV radiation [16].

Currently, there is limited information concerning the use of SLE in combination with PEF, VI, and dielectric barrier discharge cold plasma (CP) hurdles for the inhibition of melanosis and shelf life extension of postharvest PWS. The study objective was to investigate the synergistic impacts of PEF, SLE, VI, and MAP with and without CP, as innovative technologies for melanosis inhibition, quality preservation, and shelf life extension of PWS during refrigerated storage. The role of CP in combination with other hurdle technologies applied for quality preservation of Pacific white shrimp was evaluated.

## 2. Materials and Methods

### 2.1. Chemicals

Analytical-grade reagents were used, except for the fatty acid profile, for which HPLC-grade hexane was used. All chemicals were purchased from Sigma Aldrich (St. Louis, MO, USA), Merck (Damstadt, Germany), and Thermo Fisher Scientific (Auckland, New Zealand). Microbial media were bought from Himedia (Mumbai, India).

### 2.2. Preparation of SLE

Soursop leaves were obtained from a plantation located at Chana Road, Kho Hong, Hat Yai, Thailand. The leaves were washed and oven-dried at 60 °C overnight. The dried leaves were ground to powder using a high-speed grinder (Panasonic MX898N, Berkshire, UK) and sieved using 80-mesh stainless steel screen to obtain the fine powder. The SLE was prepared using a mixture of soursop leaf powder with 80% ethanol (*v/v*) at 1:10 (*w*/*v*) ratio according to the method employed by Ahmad et al. [12]. After extraction, the ethanol was evaporated (40 °C, under vacuum) using an Eyela rotary evaporator (Eyela Tokyo Rika, Tokyo Rikakikai Co., Ltd., Tokyo, Japan). The remaining ethanol was purged with N_2_, and the resulting crude extract was dechlorophyllized using the sedimentation method. The mixture of the extract and water (1:2) was kept for 24 h at 4 °C. The residue of the chlorophyll settled at the bottom of the flask, and the supernatant was collected, centrifuged (10,000× *g*, 4 °C, 30 min), frozen (−20 °C), and lyophilized. The extract powder obtained was named ‘SLE’.

### 2.3. Shrimp Collection and Preparation

PWS (freshly harvested) samples were purchased from a local market in Hat Yai, Thailand. About 50–55 shrimp per kg, with an average weight and length of 18.9 g ± 1.5 g and ~16.0 cm, respectively, were obtained. The samples were placed in a polystyrene box with ice flakes at 1:2 (*w*/*w*) ratio, in which the temperature of the sample was ~2 °C. The PWS were transported to the laboratory within 30 min.

#### 2.3.1. Preparation of PWS With and Without PEF-SLE-VI Treatments

PWS antennae were clipped with scissors. The shrimp (30 per batch) were placed in the sample chamber containing ice slurry and pretreated with PEF (PEF LAB-400 W, Febix Int’l Inc., Chiang Mai, Thailand) as detailed by Ahmad et al. [20]. Thereafter, the pretreated shrimp were drained for 5 min prior to soaking in 1% SLE solution (dissolved in distilled water) at a shrimp/extract ratio of 1:3 (*w*/*v*). The soaked shrimp were subjected to VI (pressure: 5 kPa, vacuum time: 5 min, restoration time: 5 min) for 2 consecutive cycles. Treated samples were drained for 5 min before the next processing step.

#### 2.3.2. Treatments of PWS With and Without MAP and CP

PWS samples treated with and without PEF-SLE-VI process were placed in a polystyrene plate (18 cm × 12 cm). The trays containing the PWS (6 shrimp per tray) were put in a linear low-density polyethylene (LLDPE) pouch (19 cm × 13 cm). After evacuating the air using a vacuum packing machine (AT-Packing Co. Ltd., Nonthaburi, Thailand), the packages containing the samples were filled with gases (50% CO_2_/50% Ar, 50% CO_2_/50% N_2_, or 60% CO_2_/30% N_2_/10% Ar) at a shrimp/gas ratio of 1:3 (*w*/*v*) using a gas mixer (Model KM 20–200-3 ME, WITT Co. Ltd., Nonthaburi, Thailand) and finally sealed. The control sample was filled with normal surrounding air and sealed.

The CP system was equipped with a high-voltage transformer (200 V input voltage, 50 Hz) and a voltage variac (0–200 kV output voltage) connected with a dielectric-barrier discharge system. According to the method of Shiekh and Benjakul [17], CP was generated with an output voltage of ~15 kV at atmospheric pressure. The input current and voltage characteristics were monitored by a function wave form generator Digital Storage Oscilloscope (Model DSO2D10, Hantek, Shandong, China). The upper and lower electrodes were set at a minimum of ~1.5 cm distance for effective exposure to dielectric-barrier discharge. PWS packages filled with various gas mixtures were placed between the electrodes’ gap for the treatment. All samples were subjected to CP treatment for 5 min.

Different samples were designated as follows:

1. Control (PWS packed under air without any treatment).
2. CP (PWS packed under air and subjected to CP treatment only).
3. PEF-SLE1-VI-MAP1 (PWS subjected to PEF, SLE (1%) using VI for 2 cycles, and MAP (CO_2_/Ar: 50%/50%).
4. PEF-SLE1-VI-MAP2 (PWS subjected to PEF, SLE (1%) using VI for 2 cycles, and MAP (CO_2_/N_2_: 50%/50%).
5. PEF-SLE1-VI-MAP3 (PWS subjected to PEF, SLE (1%) using VI for 2 cycles, and MAP (CO_2_/N_2_/Ar: 60%/30%/10%).
6. PEF-SLE1-VI-MAP1-CP (PWS subjected to PEF, SLE (1%) using VI for 2 cycles, MAP (CO_2_/Ar: 50%/50%), and CP.
7. PEF-SLE1-VI-MAP2-CP (PWS subjected to PEF, SLE (1%) using VI for 2 cycles, MAP (CO_2_/N_2_: 50%/50%), and CP.
8. PEF-SLE1-VI-MAP3-CP (PWS subjected to PEF, SLE (1%) using VI for 2 cycles, MAP (CO_2_/N_2_/Ar: 60%/30%/10%), and CP.

### 2.4. Analyses

#### 2.4.1. Color

Color of PWS meat was analyzed using HunterLab colorimeter (ColorFlex cx0384, Hunter associate laboratory, Inc., Reston, VA, USA) [8]. Color parameters (L*, a* and b*) were measured, and the overall color difference (ΔE*) was computed using the following formula:

$$\Delta E^{*}=\sqrt{{(L^{*}-L}^{0*}{)}^{2}+{(a^{*}-a}^{0*}{)}^{2}+{(b^{*}-b}^{0*}{)}^{2}}$$

${(L^{*}-L}^{0*}),{(a^{*}-a}^{0*}),\;and\;{(b^{*}-b}^{0*})$ represent the differences in color parameters of PWS meat during storage compared with the corresponding values on day 0.

#### 2.4.2. Melanosis Score

Degree of melanosis was assessed at 3-day intervals over the storage period by 10 trained panelists as per the method tailored by Sae-leaw and Benjakul [3]. The panelists inspected the PWS samples and recorded the scores on a scale of 0–10 (melanosis score of 0 = absent and 10 = extremely severe).

#### 2.4.3. Textural Property

Hardness and toughness of the PWS meat (at 5 ± °C) were evaluated using a TA-XT plus Texture Analyzer (Stable Micro Systems, Godalming, Surrey, UK), as detailed by Ahmad et al. [20].

#### 2.4.4. Microstructure

Microstructure of the PWS meat was visualized using a scanning electron microscope (SEM-Quanta 400, FEI, Eindhoven, The Netherlands) for both longitudinal and cross-sections. Fresh PWS (without treatment) and treated PWS samples with the longest shelf life were used for the analysis [8].

#### 2.4.5. Microbiological Quality

Microbial load of PWS samples was determined at 3-day intervals throughout the storage. Spread plate count technique was employed to enumerate the total viable count (TVC) and psychrotrophic bacteria count (PBC) on plate count agar by incubating the plates at 37 °C for 24 h and 4 °C for 10 days, respectively. Presumptive *Pseudomonas* spp. were enumerated on *Pseudomonas* isolation agar incubated at 25 °C for 48 h. Enterobacteriaceae were determined on eosin methylene blue after incubation at 37 °C for 24 h, while H_2_S-producing bacteria were inoculated on triple-sugar iron agar and incubated at 25 °C for 72 h according to the procedure of Ahmad et al. [12]. The method detailed by Ahmad et al. [8] was adopted for the determination of lactic acid-producing bacteria (LAB) and *Clostridium perfringens*. The plates of the LAB and *C. perfringens* were incubated at 37 °C for 72 h in an airtight jar, and a sachet with CO_2_ gas generator was placed inside. Black colonies grown on MRS and *Perfringens* media indicated LAB and *C. perfringens*, respectively. All samples were plated in triplicate (*n* = 3), and colonies in the range of 30–300 per plate were counted.

#### 2.4.6. pH

pH of the PWS meat was determined at 3-day intervals during the storage period following Ahmad et al.’s [13] method.

#### 2.4.7. Total Volatile Base (TVB) Content

TVB was extracted from PWS meat using trichloroacetic acid (TCA) solution (4 g/100 mL) as per the method of Sae-leaw and Benjakul [3]. Conway micro-diffusion technique was employed for the TVB content determination [27]. The results were computed and presented as mg N/100 g shrimp meat.

#### 2.4.8. Peroxide Value (PV) and Thiobarbituric Acid Reactive Substances (TBARSs)

PV of PWS meat was analyzed as described by Ahmad et al. [8]. After reaction, the absorbance was read at 500 nm using a spectrophotometer (UV-1900i model, Shimadzu, Kyoto, Japan), and the PV was computed and expressed as mg cumene hydroperoxide per kg shrimp meat. A standard curve of cumene hydroperoxide (0.5 to 2 ppm) was plotted.

TBARS value of PWS meat was determined following the method of Sae-leaw and Benjakul [3]. After reaction, the absorbance was read at 532 nm. TBARS values were computed and presented as mg MDA/kg shrimp meat. A standard curve was prepared using 0–2 ppm malonaldehyde.

#### 2.4.9. Fatty Acid (FA) Profiles

FA profiles of PWS meat were determined for all samples at day 0 and for samples having the longest shelf life (TVC < 10^6^ CFU/g). Firstly, lipid was extracted from meat using the Bligh and Dyer [28] method. Fatty acid methyl esters (FAMEs) of extracted lipid were prepared [8]. The FAMEs were injected into the gas chromatography (Agilent GC 7890B, Santa Clara, CA, USA) equipped with flame ionization detector (FID). Front inlet temperature was set at 250 °C; initial column temperature was 80 °C, which was raised to 240 °C; detector temperature was 270 °C; and column pressure was 31.62 psi. Individual FAs were detected by FID (Agilent GC 7890B, Santa Clara, CA, USA) and quantified based on the peak area. The content was reported as g/100 g lipid.

#### 2.4.10. Sensory Property

PWS (whole samples) at day 0 and day 18 of the storage whose TVC was <10^6^ CFU/g were used for the evaluation. The samples were cooked using boiling water (shrimp/boiling water ratio, 1:3 *w*/*v*, 100 ± 5 °C, 5 min) as detailed by Sae-leaw et al. [3]. Fifty untrained panelists participated for the assessment. Nine-point hedonic scale was adopted to evaluate the likeness score as per the method of Meilgaard et al. [29].

### 2.5. Statistical Analysis

Completely randomized design (CRD) was employed in the whole study, except for sensory evaluation, in which randomized completely block design (RCBD) was used. All experiments and analyses were carried out in triplicate. Analysis of variance (ANOVA) was conducted, and the mean comparison was performed using Duncan multiple comparison test. SPSS statistical software (IBM 22.0 Package, SPSS Inc., Chicago, IL, USA) was used for all statistical evaluations.

## 3. Results

### 3.1. Color Changes

**Figure 1:**
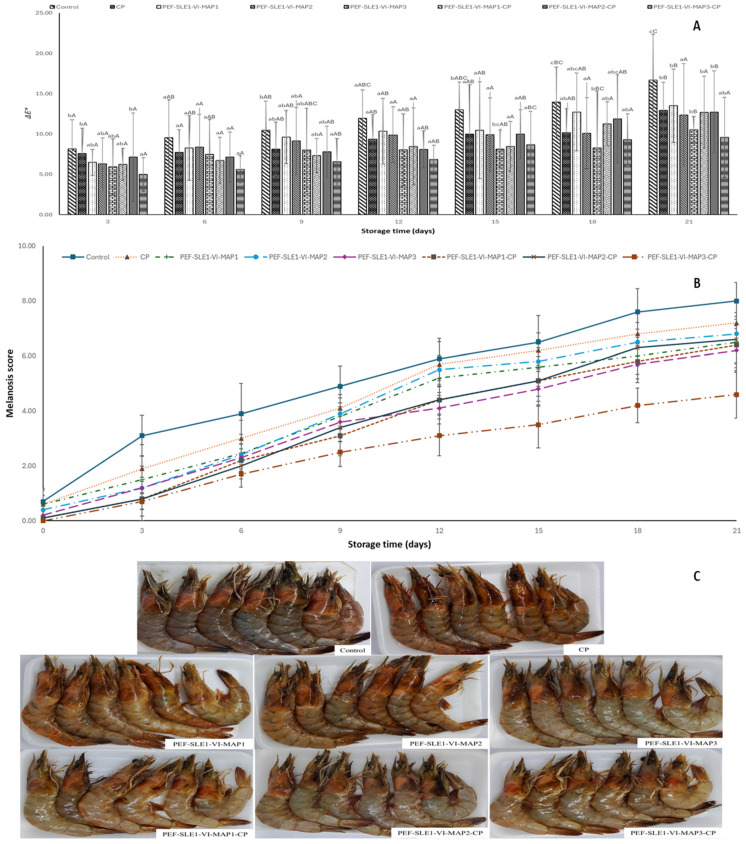
Color (**A**), melanosis (**B**), without and with different treatments during 21 days of refrigerated storage and photographs of Pacific white shrimp on day 21 (**C**). Bars represent mean ± standard deviation (*n* = 9 and 10 for color and melanosis, respectively). Control represents Pacific white shrimp packaged with normal air (without any treatment). CP represents Pacific white shrimp packaged in air and subjected to CP treatment. PEF-SLE1-VI-MAP1, PEF-SLE1-VI-MAP2, and PEF-SLE1-VI-MAP3 represent PWS subjected to PEF, SLE (1%) with the aid of VI for 2 cycles, and MAP1 (CO_2_/Ar: 50%/50%), MAP2 (CO_2_/N_2_: 50%/50%) or MAP3 (CO_2_/N_2_/Ar: 60%/30%/10%), respectively. PEF-SLE1-VI-MAP1-CP, PEF-SLE1-VI-MAP2-CP, and PEF-SLE1-VI-MAP3-CP represent PWS treated with PEF, SLE (1%) with the aid of VI for 2 cycles, and MAP1 (CO_2_/Ar: 50%/50%)-CP, MAP2 (CO_2_/N_2_: 50%/50%)-CP, or MAP3 (CO_2_/N_2_/Ar: 60%/30%/10%)-CP, respectively. Lowercase letters on the bars indicate significant differences (*p* < 0.05) within the same storage time. Uppercase letters on the bars indicate significant differences (*p* < 0.05) within the same treatment.

The color changes in the PWS treated with and without PEF, 1% SLE incorporated with the aid of VI, and subjected to MAP with and without subsequent CP treatment during 21 days of refrigerated storage are presented as the total color difference (ΔE*), as shown in Figure 1A. The ΔE* values increased with prolonged storage time. On day 3, the lowest ΔE* value (4.99 ± 2.04) was recorded in PEF-SLE1-VI-MAP3-CP (*p* < 0.05), while higher ΔE* values were observed in the control and other treated samples (*p* < 0.05). As storage progressed, ΔE* values upsurged. Among all the samples, the control, PEF-SLE1-VI-MAP1, PEF-SLE1-VI-MAP2, and CP samples showed higher increases compared with the others (*p* < 0.05). By day 21, the PEF-SLE1-VI-MAP3-CP and PEF-SLE1-VI-MAP3 samples exhibited lower ΔE* values of 9.58 ± 4.90 and 10.54 ± 1.61, respectively. Conversely, the control had the highest ΔE* values (16.67 ± 5.70). Color variations in different PWS samples were attributed to the melanosis (blackening) appearing on the shrimps’ surface, caused by PPO activity [15], and to astaxanthin (orange carotenoid pigment) degradation during storage [30]. Astaxanthin is unstable and susceptible to heat, light, oxygen, and changes in pH. Such decomposition occurred during prolonged storage [31]. The lower ΔE* values observed in the PEF-SLE1-VI-MAP3-CP and PEF-SLE1-VI-MAP3 samples resulted from the combined treatments, which lowered PPO activity. Notably, the SLE, abundant in polyphenols [13], inhibited the oxidative degradation of astaxanthin and mitigated melanosis by inhibiting the PPO activity. The gas mixtures (60% CO_2_, 30% N_2_, and 10% Ar) preserved color effectively. Higher CO_2_ levels inhibit monophenolase and diphenolase activity, reducing *o*-quinone and subsequent melanin production [9]. N_2_ is generally less reactive, but it can still promote the decomposition of muscle protein when some anaerobic bacteria grow and produce NH_3_ and some nitrogenous compounds [32]. Those microorganisms likely induce the liberation of astaxanthin. The 10% inert gas (Ar) is biochemically non-reactive, and thus, does not influence the color formation. Lower color alteration was observed in the PEF-SLE1-VI-MAP3-CP sample. Using high-voltage CP caused the ionization of the gases (CO_2_, N_2_, and Ar) to generate reactive oxygen species (ROS) such as ozone and atomic oxygen (O); reactive nitrogen species (RNS) like reactive nitrous oxide radicals (NO•), nitrate radicals (NO_2_•), nitrous acid (HNO_2_), and nitric acid (HNO_3_), which inactivate bacteria; and PPO, playing a key role in the blackening of the PWS [33,34]. The color changes in different samples were visually observed, as shown in Figure 1C. Overall, the combined treatments comprising PEF, SLE1 with the aid of VI, and MAP3-CP treatments potentially reduced discoloration in shrimp throughout the refrigerated storage period.

### 3.2. Melanosis

Melanosis scores of refrigerated PWS with and without the PEF-SLE1-VI process and MAP with and without CP during the storage are depicted in Figure 1B. On day 0, the average melanosis scores among all samples were recorded below 1.0, indicating negligible melanosis at the beginning (*p* > 0.05) among the samples. The results suggested the freshness of the samples used for the study. As the storage reached day 3, melanosis scores for the control sample increased to 3.10 ± 0.74, while the CP sample had a score of 1.90 ± 0.88. The samples treated with PEF, 1% SLE, and MAP exhibited reduced melanosis scores, ranging from 1.20 to 1.50. No differences between the PEF-SLE1-MAP2 and PEF-SLE1-MAP3 samples (*p* > 0.05) were observed. Likewise, samples treated with PEF and 1% SLE followed by CP treatment demonstrated the lowest melanosis scores (*p* < 0.05), regardless of the gas mixtures used. Beyond day 3, the melanosis scores in all samples continued to increase at different rates, depending on the treatments. On day 21, the melanosis score of the control sample attained its highest value (8.00 ± 0.67), which was significantly higher than the other treatments (*p* < 0.05). Among the samples treated with MAP, PEF-SLE1-MAP3 showed the lowest melanosis score (6.20 ± 0.79). However, the PEF-SLE1-VI-MAP3-CP sample had the lowest melanosis score (4.60 ± 0.87) among the samples treated with CP. PEF has been shown to inhibit enzyme activity by inducing conformational changes. Sheikh et al. [34] reported 40% inhibition of PWS PPO after PEF pretreatment. The higher melanosis inhibition was attained, particularly in PEF-SLE1-VI-MAP3-CP, which coincidentally showed minimal color changes, as indicated by the lowest ΔE* values (Figure 1A). This sample had the lowest melanosis at day 21 of the refrigerated storage (Figure 1C). The reduced melanosis scores were attributed to the collective individual effects of the treatments applied to the sample. The use of 1% SLE as a potent antioxidant and anti-melanosis agent alleviated the melanosis process [14]. The CP generates active species that distort the conformational structure of the PPO, thereby reducing melanosis [35,36]. The active species induced the oxidation of sensitive aromatic amino acids found in PPO, resulting in its structural modification [37]. Recently, Ahmad et al. [8] documented a lower melanosis score, minimal textural and sensorial losses, lowest microbial loads, and retarded chemical changes in PWS pretreated with PEF, 1% SLE through VI, and MAP1 (60% CO_2_, 30% N_2_, and 10% Ar) compared with other samples and the control during the 18 days of storage at 4 ᵒC. The combined effects of PEF, 2% Chamuang leaf extract solution, and high-voltage CP (Ar/air: 80/20) MAP displayed the lowest melanosis scores during 18 days of storage at 4 °C [34].

### 3.3. Textural Properties

**Figure 2:**
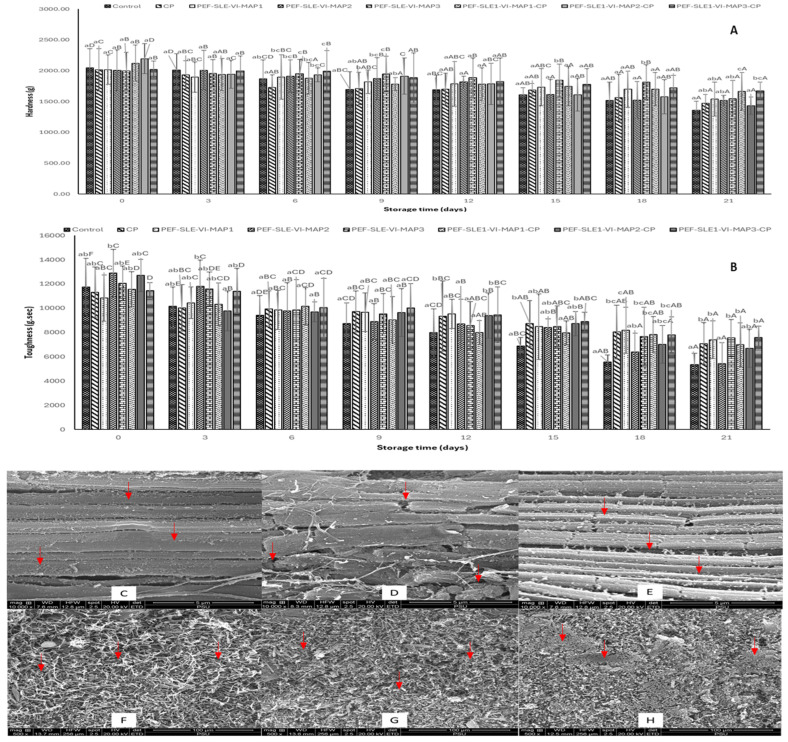
Hardness (**A**); toughness (**B**) changes in Pacific white shrimp muscles without and with different treatments during 21 days of refrigerated storage and the microstructure (longitudinal and cross-sections) of control at day 0 (**C**,**F**), PEF-SLE1-VI-MAP3 at day 18 (**D**,**G**), and PEF-SLE1-VI-MAP3-CP (**E**,**H**) at day 21. Bars represent the mean ± standard deviation (*n* = 10). Control represents Pacific white shrimp packaged with normal air (without any treatment). CP represents Pacific white shrimp packaged in air and subjected to CP treatment. PEF-SLE1-VI-MAP1, PEF-SLE1-VI-MAP2, and PEF-SLE1-VI-MAP3 represent PWS subjected to PEF, SLE (1%) with the aid of VI for 2 cycles, and MAP1 (CO_2_/Ar: 50%/50%), MAP2 (CO_2_/N_2_: 50%/50%), and MAP3 (CO_2_/N_2_/Ar: 60%/30%/10%), respectively. PEF-SLE1-VI-MAP1-CP, PEF-SLE1-VI-MAP2-CP, and PEF-SLE1-VI-MAP3-CP represent PWS treated with PEF, SLE (1%) with the aid of VI for 2 cycles, and MAP1 (CO_2_/Ar: 50%/50%)-CP, MAP2 (CO_2_/N_2_: 50%/50%)-CP, MAP3 (CO_2_/N_2_/Ar: 60%/30%/10%)-CP, respectively. Lowercase letters on the bars indicate significant differences (*p* < 0.05) within the same storage time. Uppercase letters on the bars indicate significant differences (*p* < 0.05) within the same treatment. Arrows in (**C**–**H**) indicate the observed microstructural changes.

Texture is a fundamental indicator of shrimp freshness. The major textural parameters associated with shrimp quality are hardness (firmness) and toughness. These parameters were measured every 3 days during the 21 days of refrigerated storage of the PWS samples treated with and without the PEF-1% SLE-VI process and MAP and with and without CP treatment (Figure 2). On day 0, the hardness values of both the control and treated PWS samples ranged from 1997.47 g to 2195.99 g (Figure 2A). However, no differences were found among the samples (*p* > 0.05), indicating uniformity in freshness across all samples. Also, the treatments had no immediate effect on shrimp texture. By day 3, a slight decline in hardness was noted, even though there was no noticeable difference among the various treatments and the control (*p* > 0.05). The hardness values continued to decline in all PWS samples as the storage was elongated, reaching their lowest value at day 21. The lowest hardness value (1357.47 g) at the final day of the refrigerated storage was observed in the control sample, while the PEF-SLE1-VI-MAP3-CP and PEF-SLE1-VI-MAP1-CP samples retained higher hardness values of 1674.88 g and 1664.68 g, respectively. Shrimp endogenous proteinases can migrate from the head of the shrimp to the abdomen during postmortem storage. The head of the shrimp is rich in trypsin-like proteases, which spread to the abdomen and cause hydrolysis of muscle proteins such as the myosin heavy chain, resulting in muscle softening [38]. The lower changes in hardness, especially in samples treated with the PEF-1% SLE-VI process and subjected to MAP1-CP and MAP3-CP treatments during prolonged refrigerated storage, suggested the preservation of the muscle structure. This resulted from the synergistic impact of the combined treatments. Similarly, Ahmad et al. [8] documented the preservation of textural integrity, including the firmness and toughness of PWS subjected to PEF, 1% SLE with and without VI and MAP (60% CO_2_, 10% Ar and 30% N_2_) over 18 days of refrigerated storage.

Generally, the toughness values showed a trend similar to that of the hardness. On day 0, higher toughness values were obtained for all samples, varying from 10,836.76 g.s to 12,889 g.s (Figure 2B). As the duration of the storage increased, the toughness values decreased until day 21. Notably, the control sample and PEF-SLE1-VI-MAP2 sample exhibited a significant decline in toughness (*p* < 0.05), while the other samples, particularly the PEF-SLE1-VI-MAP1, PEF-SLE1-VI-MAP3, and PEF-SLE1-VI-MAP3-CP samples, demonstrated a modest decline at the end of the storage period. The rapid deterioration of toughness in both the control and PEF-SLE1-VI-MAP2 sample was likely associated with microbial invasion [39]. The control sample was prone to microbial deterioration by spoilage microorganisms, in which structural proteins like actin and myosin were degraded for their metabolic activities [40]. Moreover, the PEF-SLE1-VI-MAP2 sample had a rapid reduction in toughness after day 6, linked to the proliferation of bacteria that exhibited the resistance to the initial treatment. The sample was treated with MAP2, which was composed of 50% CO_2_ and 50% N_2_. This composition further promoted microaerophilic and anaerobic bacterial growth. The lower toughness changes found in the PEF-SLE1-VI-MAP3-CP, PEF-SLE1-VI-MAP1, and PEF-SLE1-VI-MAP3 samples were attributed to the efficient proteases and microbial inhibition as a result of the combined hurdles. PEF pretreatment could enhance the penetration of the bioactive compounds in the SLE into the shrimp muscle, thereby preserving its integrity. The MAP1 treatment comprising 50% CO_2_ caused the inhibition of the growth of aerophiles, while 50% Ar, which is biochemically inert, maintained the toughness of shrimp muscle by replacing oxygen, which influenced lipid oxidation. MAP3 and MAP3-CP, consisting of 60% CO_2_, 30% N_2_, and 10% Ar, also enhanced toughness retention, regardless of CP treatment. Overall, the textural properties were preserved during refrigerated storage for 21 days through the combined PEF-1% SLE-VI process and MAP1 or MAP3, irrespective of CP treatment.

### 3.4. Microstructure

The microstructure of the fresh, untreated PWS muscle (control) on day 0, was compared with that of the treated PWS samples after storage but still showing the acceptable microbial limit (<6 Log CFU/g, TVC). As depicted in Figure 2C, the longitudinal section of the control sample revealed the intact striated muscle fibers made up of myofibrils arranged in parallel. The structural organization gives fresh PWS muscle elastic characteristics, contributing to its textural property [40]. At this stage, gapping was minimal as the connective tissues remained unbroken by either enzymatic or microbial activities. The longitudinal section of the muscle microstructure from the PEF-SLE1-VI-MAP3 sample, as shown in Figure 2D, displayed collapsed muscle bundles and destroyed z-disks, confirming the gapping had taken place (see arrows). The integrity of the z-disk is crucial for actin–myosin interactions [8]. The shrinkage of muscle fibers leads to crevice formation along with the degradation of connective tissues, including collagen and proteoglycans [41]. Collagen solubility in the shrimp muscle increased during refrigerated storage, indicating collagen degradation as implied by the increase in hydroxyproline content [42]. Pan et al. [43], using proteomics profiling, revealed that certain PWS muscle proteins including myosin heavy chain (MHC), α-actin, α-tropomyosin, β-tropomyosin, and myosin light chain underwent different levels of degradation during partially freezing storage for 30 days. The longitudinal section of the microstructure of PEF-SLE1-VI-MAP3-CP muscle is displayed in Figure 2E. The myofibril bundles maintained a regular parallel pattern with minimal decomposition of the muscle fibers. Nonetheless, gapping was observed to some extent, but it was less, compared with the PEF-SLE1-VI-MAP3 sample. Interestingly, the intact muscle structure of the fresh sample, as well as the loosened structure observed in the PEF-SLE1-VI-MAP3 and PEF-SLE1-VI-MAP3-CP samples, were correlated with the hardness and toughness values of the control sample at day 0 and the corresponding PEF-SLE1-VI-MAP3 and PEF-SLE1-VI-MAP3-CP samples before reaching the spoilage microbial limit (Figure 2A,B). Muscle softening is usually associated with the disappearance of the z-disks, dissociation of the actomyosin complexes, degradation of connectin, and collagen denaturation [44].

The transverse section of the fresh control PWS sample revealed sponge-like muscle bundles (Figure 2F). Shrimp muscles are primarily composed of fast-twitch fibers (high glycolytic and anaerobic) along with shorter, less densely packed sarcomeres that contribute to the fibrous texture of the muscle [45]. The extracellular matrix contains minimal connective tissue, and the cross-links formed by their collagen are not extensive, thereby limiting its toughness. Contrary to the control sample, the cross-sections of the PEF-SLE1-VI-MAP3 and PEF-SLE1-VI-MAP3-CP samples showed densely stacked muscle fibers (Figure 2G,H). This was more likely due to drip loss and muscle shrinkage during the elongated storage. This coincided with the loss in hardness and toughness during extended refrigerated storage, as highlighted in Figure 2A and Figure 2B, respectively. Overall, applying PEF-1% SLE-VI along with MAP3-CP treatment preserved the structural integrity of PWS muscle during 21 days of refrigerated storage.

### 3.5. Bacterial Load

**Figure 3:**
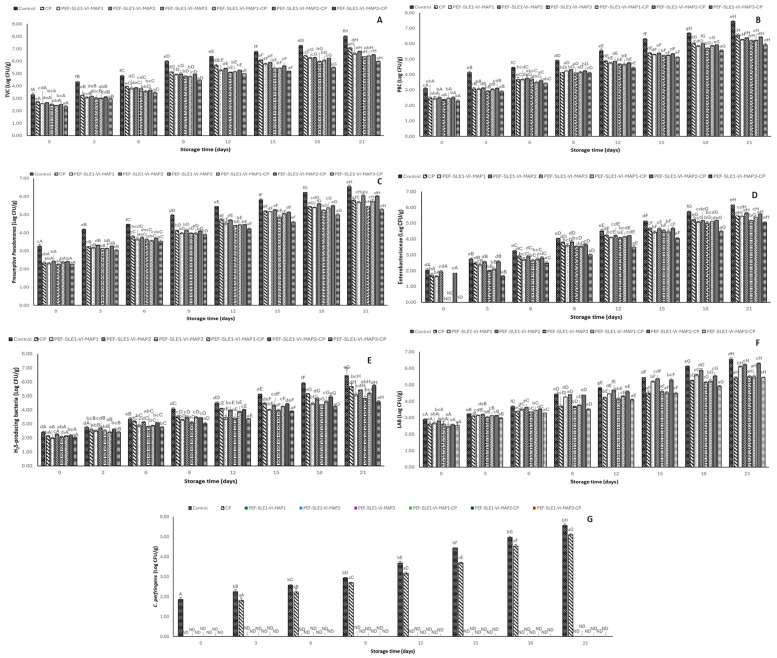
TVC (**A**), PBC (**B**), presumptive *Pseudomonas* (**C**), H_2_S-producing bacteria (**D**), Enterobacteriaceae (**E**), lactic acid bacteria (**F**), and *Clostridium perfringens* (**G**) counts in Pacific white shrimp without and with different treatments during 21 days of refrigerated storage. Bars represent the mean ± standard deviation (*n* = 3). Control represents Pacific white shrimp packaged with normal air (without any treatment). CP represents Pacific white shrimp packaged in air and subjected to CP treatment. PEF-SLE1-VI-MAP1, PEF-SLE1-VI-MAP2, and PEF-SLE1-VI-MAP3 represent PWS subjected to PEF, SLE (1%) with the aid of VI for 2 cycles, and MAP1 (CO_2_/Ar: 50%/50%), MAP2 (CO_2_/N_2_: 50%/50%), or MAP3 (CO_2_/N_2_/Ar: 60%/30%/10%), respectively. PEF-SLE1-VI-MAP1-CP, PEF-SLE1-VI-MAP2-CP, and PEF-SLE1-VI-MAP3-CP represent PWS treated with PEF, SLE (1%) with the aid of VI for 2 cycles, and MAP1 (CO_2_/Ar: 50%/50%)-CP, MAP2 (CO_2_/N_2_: 50%/50%)-CP, or MAP3 (CO_2_/N_2_/Ar: 60%/30%/10%)-CP, respectively. Lowercase letters on the bars indicate significant differences (*p* < 0.05) within the same storage time. Uppercase letters on the bars indicate significant differences (*p* < 0.05) within the same treatment.

The bacterial growth was monitored for 21 days in different refrigerated PWS samples treated with and without the PEF-1% SLE-VI process and MAP with and without CP treatment (Figure 3).

TVC, representing mesophiles, is presented in Figure 3A. At day 0, all samples showed a lower TVC, varying from 2.35 to 3.24 Log CFU/g (*p* < 0.05). The control sample (without any treatment) contained a higher bacterial load (3.24 Log CFU/g) than the other samples at this stage. The higher count likely resulted from the initial cross-contamination occurring during pre-harvest and postharvest handling. The CP-treated sample packed in air and subjected to CP treatment had a lower TVC (2.72 Log CFU/g) compared with the control sample. The reduction was attributed to the antimicrobial effect of the active species generated during the CP treatment. Other treated samples showed varying TVC on day 0, with the following ascending order: PEF-SLE1-VI-MAP3-CP < PEF-SLE1-VI-MAP1-CP < PEF-SLE1-VI-MAP3 < PEF-SLE1-VI-MAP2-CP < PEF-SLE1-VI-MAP1 < PEF-SLE1-VI-MAP2. The lower TVC in these samples resulted from the impacts of the combined treatments involving the PEF, which assists the efficiency of the antibacterial agents present in the SLE as well as the efficacy of the MAP or CP treatments on the viability of the mesophilic bacteria. PEF treatment in combination with vacuum impregnation (VI) enhanced the penetration of target compounds through the micropores created on the shrimp shells [20]. This process involves the removal of fluid occupying the pores during the vacuum impregnation process and replacing it with the solution of the bioactive compounds when ambient pressure is restored [46].

As the storage time progressed, there was an increase in TVC across all samples at different rates. The control sample showed a rapid increase in TVC as the storage time upsurged, while the PEF-SLE1-VI-MAP3-CP sample showed the lowest increase in TVC (*p* < 0.05). On day 9, the control sample reached the acceptable microbial limit (6 Log CFU/g) according to the International Commission for Microbiological Specifications for Foods (ICMSF) [47], and it surpassed the unacceptable limit (> 7 Log CFU/g) [48] on day 15. Nonetheless, the other treated samples remained below the limit. By day 21, all samples exceeded the microbial limit, except PEF-SLE1-VI-MAP3-CP, in which TVC was still below the limit. The role of PEF in bacterial inactivation involves creating micropores on the surface of the cell membrane of the biological material through an electroporation mechanism, resulting in permeabilization of the bacterial cell membrane and causing the leakage of cellular material. Additionally, PEF pores created on the shrimp shells’ surface enhanced the migration of the active compounds in SLE that were infused into the shrimp body through VI means. The SLE components penetrate the permeable bacterial cell membrane and cause the disruption of the metabolic activities of the cell, which eventually leads to cell death. The active species that could be generated from the CO_2_, N_2_, and Ar found in MAP3-CP likely include ROS such as O_3_ and singlet O_2_ from CO_2_ ionization [49]. Additionally, H_2_O_2_ and OH• from the ionization of moisture on the shrimp surface or from the condensed water vapor in the packaging; RNS, including NO_3_^−^, NO_2_^−^, and NO; and the inert Ar excited into active Ar atoms; play a major role in inhibiting microbial growth [50,51]. Secondary reactions and recombinations between generated species occur to generate compounds like nitrous acid and carbonic acid, which have antimicrobial functions and preservative roles [52]. Mittal et al. [53] documented that PWS pretreated with PEF and soaked in 2% chitooligosaccharide–catechin conjugate solution with the aid of VI reduced melanosis, TVB-N, TMA, PV, and TBARS after 15 days of storage at 4 °C. Additionally, the total bacterial counts, including the psychrotrophic bacteria, *Pseudomonas* spp., H_2_S-producing bacteria, and Enterobacteriaceae were lower, compared with other samples [53].

The PBC of PWS samples during 21 days of refrigerated storage with and without the PEF-1% SLE-VI process and MAP and with or without CP treatment is illustrated in Figure 3B. On day 0, PBC ranged from 2.30 to 3.09 Log CFU/g, in which the control sample had a significantly higher count than the treated samples (*p* < 0.05). No substantial variations were noted among the treated samples (*p* > 0.05), except for PEF-SLE1-VI-MAP3-CP and PEF-SLE1-VI-MAP3 samples, which exhibited lower counts (*p* < 0.05). As the storage proceeded, PBC upsurged steadily among all the PWS samples, especially in the control sample, which increased at a faster rate compared with the other treatment groups. At the end of the refrigerated storage, the control sample reached 7.41 Log CFU/g. The accelerated growth in psychrotrophic bacteria in the control sample resulted from the lack of inhibitory hurdles, as the refrigerated temperature (4 °C ± 1) is optimal for their growth [54]. Conversely, the samples subjected to the combined treatments, particularly the PEF-SLE1-VI-MAP3-CP sample, had a slower increase in PBC throughout the storage.

Presumptive *Pseudomonas* spp. counts (PPC) in different shrimp samples are depicted in Figure 3C. The initial *Pseudomonas* spp. loads ranged from 2.25 to 3.26 Log CFU/g (*p* < 0.05). The control sample had a higher initial count, likely due to initial contamination and the absence of any pretreatment. The PEF-SLE1-VI-MAP3-CP sample exhibited the lowest count, probably as a result of the combined hurdles applied, which possibly reduced the level of the initial bacterial load. Spoilage of seafoods such as shrimp has been associated with *Pseudomonas* spp. as a major specific spoilage microorganism [55]. There was an upsurge in the PPC among all the samples as the storage length persisted. The rate of PPC increase varied from sample to sample. By day 21, most of the samples were heavily loaded with *Pseudomonas* spp., which likely played a dominant role in spoilage. The control sample showed the highest PPC at 6.54 Log CFU/g, followed by the PEF-SLE1-VI-MAP2 and PEF-SLE1-VI-MAP2-CP samples, respectively. This suggested that the multi-hurdle treatments comprising the PEF-1% SLE-VI process and MAP2, with or without CP, had only a short-term temporary impact against the presumptive *Pseudomonas* spp. population. The bacteria were able to recover from the stress during the treatments. They developed a resistance mechanism, allowing them to continue growing during prolonged refrigerated storage. MAP2 gas composition, whether the intact or ionized form known as cold plasma active species (MAP2-CP), appeared to have a minimal suppressive impact on *Pseudomonas* spp., making it less effective at preventing their proliferation. In contrast, the PEF-SLE1-VI-MAP3 and PEF-SLE1-VI-MAP3-CP samples demonstrated lower PPCs on day 21. The synergistic effects of the hurdle treatments involving PEF at 800 pulses, 1% SLE soaking with the aid of VI, and MAP3 gases, especially when ionized to active species in MAP3-CP, mitigated the growth of presumptive *Pseudomonas* spp. during refrigerated storage.

The presence of Enterobacteriaceae spp. in different refrigerated PWS samples during the 21 days of storage is shown in Figure 3D. On day 0, the Enterobacteriaceae spp. counts were significantly varied between 0 and 2.03 Log CFU/g (*p* < 0.05). The control sample exhibited a higher count (2.03 Log CFU/g), followed by the PEF-SLE1-VI-MAP2, PEF-SLE1-VI-MAP2-CP, CP, and PEF-SLE1-VI-MAP1 samples, respectively. The Enterobacteriaceae were not detected in the PEF-SLE1-VI-MAP3, PEF-SLE1-VI-MAP1-CP, and PEF-SLE1-VI-MAP3-CP samples on day 0. Enterobacteriaceae is a large family of bacteria that are known to be pathogenic and may cause spoilage. The most common Enterobacteriaceae genera found in seafoods include *Escherichia*, *Klebsiella*, *Salmonella*, *Enterobacter*, and *Proteus* [56,57]. The lower count and absence of growth in some PWS samples indicated minimal contamination, probably due to good hygienic practices employed during postharvest handling as well as the effectiveness of the treatment combination against the bacteria. As the storage reached day 3, all samples displayed different levels of Enterobacteriaceae loads. The control sample exhibited the highest level, compared with the other treated samples. At the same time, the lowest count was observed in the PEF-SLE1-VI-MAP3-CP sample. Similar trends were observed until the end of the storage period. On day 21, the Enterobacteriaceae level reached 6.15 Log CFU/g for the control sample, followed by 5.64 Log CFU/g for the PEF-SLE1-VI-MAP2 sample, while the PEF-SLE1-VI-MAP3 and PEF-SLE1-VI-MAP3-CP samples had lower counts. Enterobacteriaceae, as Gram-negative bacteria, are susceptible to PEF, which creates nanopores in their cell membranes [58]. The effect involves destroying the membrane integrity, causing leakage of cellular contents and reducing cell viability [59]. Also, PEF enhanced the permeability of the cell, allowing deeper penetration of antibacterial compounds in SLE with the aid of VI, thereby disrupting ATP production, DNA replication, and causing mitochondrial dysfunction [60]. The ROS, RNS, and forms of active species generated through CP, particularly when MAP3 gases were involved, likely induced oxidative stress to the lipids, proteins, and DNA of the bacterial cell, leading to its inactivation.

H_2_S-producing bacteria counts of PWS samples with different treatments are presented in Figure 3E. On day 0, the H_2_S-producing bacteria counts were between 2.0 and 2.41 Log CFU/g (*p* < 0.05). The control sample showed a higher count than all the treated groups. As the storage continued up to day 21, there was a continuous upsurge in the level of H_2_S-producing bacteria for all the samples, but the rate of increase was varied. The increasing H_2_S-producing bacteria count, particularly in the control sample, was plausibly due to the ability of the bacteria to thrive on the shrimp sample as a result of the availability of nutrients and the absence of any hurdle to impede their growth. Treating PWS with the PEF-1% SLE-VI process and then subjecting them to MAP, particularly using MAP3, followed by CP treatment (PEF-SLE1-VI-MAP3-CP sample) resulted in the retardation of H_2_S-producing bacterial growth. H_2_S-producing bacteria associated with shrimp spoilage include *Pseudomonas* spp., *Shewanella* spp., *Vibrio* spp. [61,62], and *Clostridium* spp. Thus, their inactivation mechanism is similar to that of the Enterobacteriaceae and presumptive *Pseudomonas* spp. discussed previously.

LAB counts of refrigerated PWS samples with varying treatments during the storage are presented in Figure 3F. On the first day of the refrigerated storage, the LAB count in the control sample (2.91 Log CFU/g) was higher (*p* < 0.05) than in the other samples. Among the treated groups, the PEF-SLE1-VI-MAP2 sample had the highest LAB count (2.82 Log CFU/g), whereas the lowest level (2.49 Log CFU/g) was obtained in the PEF-SLE1-VI-MAP1-CP sample (*p* < 0.05). No noticeable variations in LAB counts were observed among the PEF-SLE1-VI-MAP1, PEF-SLE1-VI-MAP3, PEF-SLE1-VI-MAP2-CP, and PEF-SLE1-VI-MAP3-CP samples on day 0 (*p* > 0.05). Increased levels of LAB were witnessed in all the PWS samples as the storage time progressed. A marked increase was found in the later stage of the storage period. Until the final day, the PEF-SLE1-VI-MAP3-CP, PEF-SLE1-VI-MAP1-CP, PEF-SLE1-VI-MAP3, and CP samples maintained lower levels of LAB count among the treated groups, and no differences existed among them (*p* > 0.05) on day 21. LAB, the microaerophiles, continued to proliferate in all the samples, likely due to the absence of any treatment for the control sample to mitigate their growth. Lower levels of LAB were exhibited throughout the storage period in all CP-treated samples except the PEF-SLE1-VI-MAP2-CP sample. These results suggested the effectiveness of the CP integration to cause the inactivation of LAB through generated active species and the complemented impacts of the antibacterial property of 1% SLE through VI. On the other hand, higher levels of LAB were observed in samples without CP treatment (except PEF-SLE1-VI-MAP3, which maintained lower levels). This was attributed to the ability of LAB to survive in anaerobic conditions. Although LAB could contribute to shrimp spoilage, they might also complement shrimp preservation through different mechanisms. LAB produce antimicrobial compounds such as lactic acid and bacteriocins, which perhaps acted as antagonists to certain spoilage bacteria, including *Pseudomonas* and *Shewanella*, as well as pathogenic bacteria, such as *Vibrio* and *Listeria*, associated with seafoods [63,64,65].

*C. perfringens* growth was monitored in the PWS samples with varying treatments (Figure 3G). On day 0, only the control sample had *C. perfringens* count at 1.86 Log CFU/g, whereas no growth was noticed among all the treated groups. On day 3, the control and CP samples displayed *C. perfringens* loads at 2.24 and 1.81 Log CFU/g, respectively. No colonies were detected in the other samples (*p* < 0.05). During extended storage, the *C. perfringens* increased gradually in these two samples (control and CP sample), and the control sample demonstrated higher counts than the CP. On day 21, both the control and the CP samples reached their highest levels, 5.56 and 5.10 Log CFU/g, respectively. Conversely, no count was detected in the other treated samples until the end of the storage period. *C. perfringens* is a Gram-positive, anaerobic, and spore-forming bacterium and is known for causing foodborne gastroenteritis, especially in the low or oxygen-deficient modified atmosphere packaged foods [65]. However, its growth in the refrigerated PWS sample was hindered by the hurdles involved during preparation and storage. *C. perfringens* is known to be a mesophilic bacterium, but some strains have the potential to grow below ambient temperature. Storing the sample at 4 °C annihilated its growth to some level. PEF pretreatment likely had a lower impact on *C. perfringens* due to its thick, multilayered peptidoglycan cell wall. However, due to the micro/nanopores created on the PWS surface through the PEF as a result of electroporation, the muscle absorbed the antibacterial SLE components with the aid of VI. Additionally, the samples treated with MAP, without and with CP and irrespective of the gas compositions, demonstrated strong growth inhibition of *C. perfringens,* plausibly by complementing other hurdles. Overall, the combined hurdles, such as the PEF-1% SLE-VI process and MAP, especially MAP3 followed by CP treatment, inhibited the growth of most of the spoilage and pathogenic bacteria in PWS during the 21-day refrigerated storage.

### 3.6. pH

**Figure 4:**
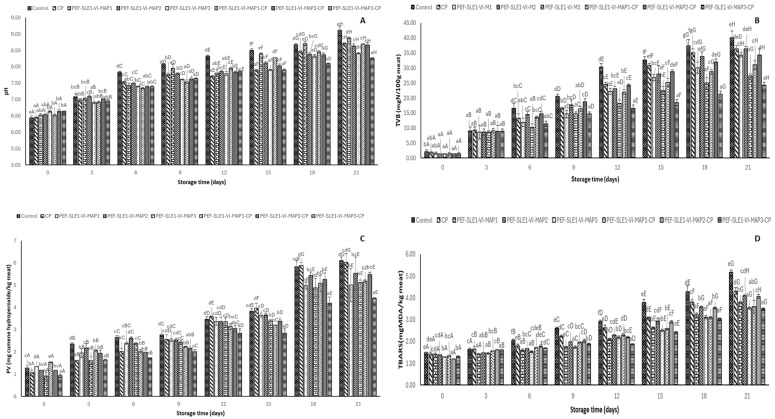
pH (**A**), total volatile basic content (**B**), peroxide value (**C**), and thiobarbituric acid reactive substances (TBARS) (**D**) of Pacific white shrimp without and with different treatments during 21 days of refrigerated storage. Bars represent the mean ± standard deviation (*n* = 3). Control represents Pacific white shrimp packaged with normal air (without any treatment). CP represents Pacific white shrimp packaged in air and subjected to CP treatment. PEF-SLE1-VI-MAP1, PEF-SLE1-VI-MAP2, and PEF-SLE1-VI-MAP3 represent PWS subjected to PEF, SLE (1%) with the aid of VI for 2 cycles, and MAP1 (CO_2_/Ar: 50%/50%), MAP2 (CO_2_/N_2_: 50%/50%), or MAP3 (CO_2_/N_2_/Ar: 60%/30%/10%), respectively. PEF-SLE1-VI-MAP1-CP, PEF-SLE1-VI-MAP2-CP, and PEF-SLE1-VI-MAP3-CP represent PWS treated with PEF, SLE (1%) with the aid of VI for 2 cycles, and MAP1 (CO_2_/Ar: 50%/50%)-CP, MAP2 (CO_2_/N_2_: 50%/50%)-CP, or MAP3 (CO_2_/N_2_/Ar: 60%/30%/10%)-CP, respectively. Lowercase letters on the bars indicate significant differences (*p* < 0.05) within the same storage time. Uppercase letters on the bars indicate significant differences (*p* < 0.05) within the same treatment.

The pH of the PWS samples subjected to various treatments during 21 days of refrigerated storage is presented in Figure 4A. The pH of all samples at day 0 ranged from 6.44 to 6.64. There was no significant variation between the control and other treated samples (*p* > 0.05). The slightly lower initial pH recorded in all samples was within the normal pH range of the fresh PWS sample, indicating the samples’ freshness. However, the slightly lower pH of PWS could be as a result of the initiation of postmortem glycolysis mediated by endogenous enzymes, which converts glycogen to lactic acid within a few hours after death. On day 3, there was an increase in pH among all samples, whereby the control, PEF-SLE1-VI-MAP1, PEF-SLE1-VI-MAP2, and PEF-SLE1-VI-MAP2-CP samples showed higher pH, but no differences in pH values were obtained among the samples (*p* > 0.05). A lower pH increase was also noticed among CP, PEF-SLE1-VI-MAP3, PEF-SLE1-VI-MAP1-CP, and PEF-SLE1-VI-MAP3-CP samples, but no noticeable variation was found among those samples (*p* > 0.05). This trend was extended until day 12 of the storage period. The pH increased at a faster rate among all samples from day 15 to day 21. The control sample exhibited the highest increase, with the pH reaching 9.12 on day 21, while the PEF-SLE1-VI-MAP3-CP sample demonstrated the lowest pH increase within the corresponding storage days. The increase in pH was associated with the increase in psychrotrophic bacteria, comprising *Pseudomonas*, *Shewanella* etc. (Figure 2A), as well as the rise in presumptive *Pseudomonas* spp. (Figure 2B). Typically, the elevated pH resulted in the bacterial deamination of the amino acids in shrimp to release ammonia as well as the conversion of the trimethylamine oxide (TMAO) abundant in shrimp into trimethylamine (TMA) [66]. Additionally, the autolytic process arising from the shrimp tissue proteases perhaps broke down the proteins into free amino acids, which were further deaminated into ammonia and other volatile basic compounds [67]. The lower pH variation observed in the samples treated with the PEF-1% SLE-VI process and subsequent MAP, especially using MAP3 ionized by CP, was possibly related to the reduced microbial growth over the storage period. Hence, the combined treatments lowered the pH changes during the 21-day refrigerated storage period.

### 3.7. Total Volatile Basic Content (TVB)

TVB contents in PWS samples with varying treatments during 21 days of refrigerated storage are presented in Figure 4B. On day 0, the TVB content varied between 2.10 and 1.40 mg N/100g meat, and no variation was recorded among the treated samples (*p* > 0.05), indicating the uniformity of the samples used for the study. On day 3, there was an upsurge in the TVB values among all the samples, even though no variations were recognized among both the treated groups and the control samples (*p* > 0.05). When the storage reached 6 days, there was a further increase in the TVB values at different levels, but the control sample showed the highest value (16.57 mg N/100g meat), followed by the PEF-SLE1-VI-MAP2 and PEF-SLE1-VI-MAP2-CP samples, respectively. Lower TVB values were observed in the PEF-SLE1-VI-MAP3 and PEF-SLE1-VI-MAP3-CP samples. Similar trends were found in the subsequent storage time. The control sample attained the recommended acceptable limit for TVB in seafoods (30 mg N/100g meat) [7] up to day 12 of refrigerated storage, while all the treated samples were still below the limit on the same day. On day 21 of the refrigerated storage, all the samples exceeded the acceptable TVB limit except for the PEF-SLE1-VI-MAP3 and PEF-SLE1-VI-MAP3-CP samples. TVB value has been an important spoilage indicator and can be associated with the sensorial and microbiological quality of seafoods. Certain spoilage bacteria in seafoods, commonly *Pseudomonas*, *Shewanella*, and *Aeromonas* spp., are associated with seafood spoilage [68]. The TMAO available in shrimp was reduced to ammonia, trimethylamine, dimethylamine, etc., collectively known as volatile basic compounds and quantified as TVB-N content [69]. When the volatile basic compounds generated exceed the allowable limit, seafood is not suitable for consumption and is associated with several health risks caused by microorganisms or their toxins. The higher TVB-N value found in the control resulted from the non-treatment carried out on the sample to mitigate microbial and enzymatic activities during the storage period. The PEF-SLE1-VI-MAP2 and PEF-SLE1-VI-MAP2-CP samples underwent similar treatments except for the CP involved in the latter. The gas composition (50% CO_2_ and 50% N_2_) as well as the active species generated, such as ROS and RNS, lowered the growth of some spoilage bacteria but simultaneously encouraged the proliferation of others, which supported the production of volatile basic compounds. The retarded TVB-N contents observed during the storage period, especially in the PEF-SLE1-VI-MAP3-CP sample, emanated from the synergistic impacts of PEF, which inactivated some spoilage bacteria and endogenous enzymes and enhanced the penetration of the bioactive compounds present in the 1% SLE solution, thus inhibiting the growth of the spoilage bacteria. Furthermore, the application of MAP, particularly MAP3, followed by CP treatment led to the surface decontamination of the spoilage bacteria on the shrimp surface and the packaging.

### 3.8. Peroxide Value (PV) and Thiobarbituric Acid Reactive Substances (TBARS)

Changes in the PVs of refrigerated PWS samples subjected to different treatments are presented in Figure 4C. On day 0, the PVs of all samples ranged from 0.92 to 1.54 mg cumene hydroperoxide/kg meat. There was variation in PVs among the samples (*p* < 0.05). The variations in PVs among samples at day 0 were connected to the individual sample differences. On day 3, a higher PV was noticed in the control sample, preceded by the PEF-SLE1-VI-MAP2 and PEF-SLE1-VI-MAP2-CP samples, whereas the CP, PEF-SLE1-VI-MAP3-CP, and PEF-SLE1-VI-MAP3 samples showed lower PVs. The variations in PVs increased as the storage period was lengthened, and the sample-to-sample variations were widened through 15 days of storage. On day 18, there was a dramatic increase in the PVs of all samples. However, on day 21, there was no increase in the PVs across any of the samples. Possibly, the higher initial PVs recorded in some treated samples during the early days of storage were influenced by the PEF and CP treatments, which promoted the leakage of lipase from the shrimp tissue membranes or bacterial cells, thereby initiating lipid oxidation manifested in the later stages of the storage period [70,71]. In contrast, the lower PVs were observed, particularly in the PEF-SLE1-VI-MAP3-CP sample, throughout the storage period. The combined treatment likely played a significant role in retarding the primary lipid oxidation. Although the PEF and CP treatments carried out on the sample enhanced the generation of peroxides, such a drawback was tackled by the antioxidant phenolic compounds abundant in the SLE solution. Phenolic compounds likely scavenged the free radicals and peroxyl radicals generated. The choice of MAP3-CP devoid of oxygen and with higher CO_2_ and inert gases decreased the oxidation process. PWS samples pretreated with PEF and then immersed in 2% Chamuang leaf extract followed by high-voltage cold atmospheric plasma treatment on Ar/air mixture (80:20) exhibited the lowest PV during 18 days of refrigerated storage [17].

The TBARS values of refrigerated PWS samples subjected to various treatments are presented in Figure 4D. On day 0, TBARS values among all samples ranged from 1.20 to 1.49 mg MDA/kg meat. The variations in TBARS values resulted from the slight disparities between the samples. Minor increases in the TBARS values were noticed among all PWS samples as the storage proceeded to days 3 and 6. This was attributed to the retarded secondary oxidation process, plausibly caused by the multi-targeted impacts involving the lower storage temperature (4 °C), which reduced the activities of the lipoxygenases. Hindrance by the antioxidant phenolic compounds from the 1% SLE solution, as well as the MAP being deficient in oxygen, lowered the lipid oxidation. From day 12 to 21, there was a gradual increase in TBARS values, which was more pronounced in the control and CP samples. This coincided with higher PV values recorded during the extended storage period (Figure 4C). The peroxides produced, which were unstable, were likely decomposed to secondary oxidation products such as malondialdehydes [72]. On the other hand, treated groups including the PEF-SLE1-VI-MAP1, PEF-SLE1-VI-MAP3, PEF-SLE1-VI-MAP1-CP, and PEF-SLE1-VI-MAP3-CP samples demonstrated lower increases in the TBARS values during the elongated storage. This was perhaps associated with the combined treatments, especially the antioxidant agents in SLE. Several polyphenols, including kaemferol-3-O-rutinoside, catechin, chlorogenic acid, ferulic acid, coumaric acid, gallic acid, vanillic acid, 4-hydroxybenzoic acid, ellagic acid, trans-cinnamic acid, epigallocatechin, rutin, and caffeic acid, have recently been identified in SLE [12,13]. Hence, the multi-hurdle approach involving the PEF-1%SLE-VI process and MAP followed by CP retarded the formation of secondary lipid oxidation products in PWS samples for 21 days under refrigeration.

### 3.9. Fatty Acid Profiles

The fatty acid profiles of all PWS samples on day 0 of the refrigerated storage, in comparison with the samples that exhibited lower lipid degradation (low PV and TBARS) after 21 days of refrigerated storage, are tabulated in Table 1. Samples on day 0 contained saturated fatty acids (SFAs), monounsaturated fatty acids (MUFAs), and polyunsaturated fatty acids (PUFAs). Some fatty acids were present in low amounts in some samples and not detected in others, likely due to individual sample variations. Among the SFAs, stearic acid and butyric acid were detected in higher amounts in all samples. The control sample exhibited the lowest quantity of butyric acid (15.51 g/100 g), followed by PEF-SLE1-VI-MAP3-CP (28.28 g/100 g), PEF-SLE1-VI-MAP3 (31.101 g/100g), and CP (31.48 g/100 g). The higher amount of butyric acid was observed in PEF-SLE1-VI-MAP1 (42.07 g/100 g), PEF-SLE1-VI-MAP1-CP (41.37 g/100 g), PEF-SLE1-VI-MAP2 (40.92 g/100 g), and PEF-SLE1-VI-MAP2-CP (40.69 g/100 g), respectively. The reduced butyric acid level in the control sample might be due to the fact that the lipid content was unaltered, while samples subjected to PEF and CP treatments were exposed to oxidation, depending on the conditions of the exposure [71,72]. For stearic acid, the control showed the highest quantity, followed by CP, while the lowest quantity was observed in PEF-SLE1-VI-MAP3, followed by PEF-SLE1-VI-MAP3-CP (*p* < 0.05). The total PUFAs, majorly comprising docosahexaenoic (DHA), eicosapentaenoic (EPA), and linoleic acids, showed higher values in the PEF-SLE1-VI-MAP3 and PEF-SLE1-VI-MAP3-CP samples, indicating minimal alteration of the PUFAs in those treatments, compared with others. Additionally, the PEF-SLE1-VI-MAP3-CP sample had the lowest amount of total SFAs (*p* < 0.05) among all the samples on day 0, indicating a reduced level of oxidation. The variations were perhaps associated with the treatments involved during preparation. Coincidentally, a similar trend was observed in the PVs of the aforementioned samples on day 0 (Figure 4C), highlighting the correlation between the peroxide levels with the composition of fatty acids and their degree of saturation [8].

On day 21, the SFA levels increased, while the levels of MUFAs and PUFAs were reduced (*p* < 0.05) in both the PEF-SLE1-VI-MAP3 and PEF-SLE1-VI-MAP3-CP samples when compared with the corresponding samples on day 0. However, the PEF-SLE1-VI-MAP3-CP sample demonstrated a smaller increase in total SFAs than the PEF-SLE1-VI-MAP3 counterpart. This resulted from the combined effects involving the PEF and CP to inactivate the lipoxygenase, the antioxidant activity associated with the polyphenols present in the 1% SLE solution, as well as the impact of the selected MAP3 deficient in oxygen. Hence, oxidation of unsaturated fatty acids in shrimp was minimized during refrigerated storage via the combined hurdles involving the PEF-1% SLE-VI process and the MAP3 followed by CP treatment.

### 3.10. Sensory Property

Likeness scores of the cooked PWS samples treated without and with the PEF-1% SLE-VI process and MAP without and with CP treatment on day 0, in comparison with the selected sample having lower microbial load on day 18 of the refrigerated storage, are presented in Table 2. On day 0, all samples received higher likeness scores in all the sensory attributes evaluated. There was no variation observed among the treated samples and the control group (*p* > 0.05). This indicated that the treatments employed were nondestructive as the sensory parameters were not compromised. Comparatively, higher likeness scores of PWS samples were observed at day 0 for several attributes such as the appearance, color, texture, and odor. The result agreed with those of the respective melanosis scores and instrumental color (Figure 1A–C), hardness and toughness (Figure 2A,B), and the TVB content (Figure 4B) of the corresponding samples on the same day. As the storage reached day 18, the PEF-SLE1-VI-MAP3-CP sample was selected for sensory evaluation due to its lower microbial load to ensure the safety of the panelists or consumers. All sensory attributes received ‘moderately acceptable’ ratings by the panelists, with an overall score of ‘6.4’, indicating acceptability of the sample. However, the result indicated a noticeable variation (*p* < 0.05) from the score of the same on day 0. The lower sensorial changes were attributed to the combined treatments employed on the sample, including the PEF and VI treatments, which aided the infusion of the SLE components acting as preservative. The MAP3 followed by CP treatment had multi-targeted functions such as decontaminating the sample and the container from spoilage microorganisms and contaminants. Additionally, the selected gases prevented the oxidation and minimized interaction with the sample.

## 4. Conclusions

The combination of PEF, 1% SLE through VI, and MAP3 (60% CO_2_, 30% N_2_, and 10% Ar) followed by CP treatment effectively preserved the quality and extended the shelf life of Pacific white shrimp (PWS) during refrigerated storage. These treatments significantly reduced melanosis, preserved color and texture, and lowered microbial growth, compared with the control. Notably, the PEF-SLE1-VI-MAP3-CP sample demonstrated the preserved muscle structure and substantial reduction in spoilage bacteria growth while maintaining lower pH and total volatile base content. The antioxidant property of SLE mitigated lipid oxidation. The shelf life of the PWS sample with the most effective treatment (PEF-SLE1-VI-MAP3-CP) was extended up to 21 days during refrigerated storage, while sensorial acceptability still existed. These findings highlight the potential of integrating nonthermal technologies with plant extracts as a means for seafood preservation. Although the hurdle technology could be expensive due to the high initial cost of the equipment and skilled labor, the operating cost and time could drastically be reduced through process control and automation. Thus, the effective implementation of combined nonthermal processing technologies and plant extracts could significantly reduce the substantial economic losses associated with quality deterioration in the shrimp industry.

## Figures and Tables

**Table 1:** Fatty acid profiles of Pacific white shrimp treated without and with PEF-1% SLE-VI process and MAP without and with CP treatment on day 0 and the selected treated samples stored at 4 °C for 21 days.

Fatty Acid (g/100g Lipid)	Control	Day 0						Day 21		
		CP	PEF-SLE1-VI-MAP1	PEF-SLE1- VI-MAP2	PEF-SLE1- VI-MAP3	PEF-SLE1- VI-MAP1-CP	PEF-SLE1- VI-MAP2-CP	PEF-SLE1-VI- MAP3-CP	PEF-SLE1- VI-MAP3	PEF-SLE1- VI-MAP3-CP
C4:0 (Butyric)	15.51 ± 2.63 ^a^	31.48 ± 4.04 ^b^	42.07 ± 3.91 ^c^	40.92 ± 7.43 ^c^	31.10 ± 0.91 ^b^	41.37 ± 2.19 ^c^	40.69 ± 5.30 ^c^	28.28 ± 4.24 ^ab^	43.71 ± 3.00 ^c^	37.86 ± 4.42 ^b^
C12:0 (Lauric)	0.56 ± 0.04	ND	ND	ND	ND	ND	ND	ND	ND	ND
C14:0 (Myristic)	0.57 ± 0.03 ^b^	ND	ND	0.45 ± 0.00 ^a^	ND	ND	ND	ND	ND	0.36 ± 0.01 ^a^
C15:0 (Pentadecanoic)	ND	ND	0.31 ± 0.01 ^a^	0.53 ± 0.13 ^b^	0.39 ± 0.09 ^a^	0.49 ± 0.11 ^a^	0.34 ± 0.08 ^a^	0.46 ± 0.08 ^a^	0.56 ± 0.20 ^b^	0.4 ± 0.00 ^a^
C16:0 (Palmitic)	16.64 ± 0.98 ^b^	16.04 ± 1.51 ^b^	10.87 ± 0.91 ^a^	ND	ND	ND	ND	ND	ND	ND
C17:0 (Heptadecanoic)	2.16 ± 0.31 ^b^	2.00 ± 0.00 ^b^	1.14 ± 0.23 ^a^	2.27 ± 0.43 ^b^	1.85 ± 0.25 ^ab^	12.4 ± 0.19 ^c^	1.63 ± 0.16 ^ab^	2.06 ± 0.58 ^b^	2.03 ± 0.62 ^b^	1.81 ± 0.45 ^a^
C18:0 (Stearic)	9.03 ± 0.71 ^c^	8.65 ± 0.90 ^c^	5.77 ± 0.47 ^a^	6.68 ± 0.91 ^ab^	6.66 ± 0.93 ^ab^	7.25 ± 0.22 ^b^	6.09 ± 0.66 ^a^	6.48 ± 1.94 ^ab^	7.11 ± 0.87 ^b^	7.87 ± 0.89 ^b^
C20:0 (Arachidic)	ND	11.42 ± 1.25 ^c^	7.83 ± 0.61 ^a^	9.40 ± 0.66 ^b^	ND	ND	ND	ND	ND	ND
C14:1 (Myristoleic)	ND	ND	ND	ND	0.31 ± 0.00 ^a^	0.33 ± 0.01 ^a^	ND	ND	ND	0.33 ± 0.00 ^a^
C15:1 cis 10 (Pentadecanoic)	17.97 ± 1.06 ^c^	12.04 ± 1.60 ^a^	13.05 ± 0.65 ^b^	12.17 ± 1.76 ^a^	13.51 ± 1.43 ^b^	13.39 ± 0.43 ^b^	11.55 ± 1.26 ^a^	15.70 ± 4.16 ^b^	12.08 ± 3.99 ^a^	14.30 ± 0.54 ^b^
C16:1 (Palmitoleic)	0.91 ± 0.04 ^c^	0.89 ± 0.00 ^c^	0.64 ± 006 ^a^	0.66 ± 0.07 ^a^	0.74 ± 0.09 ^b^	0.77 ± 0.02 ^b^	0.68 ± 0.09 ^a^	0.67 ± 0.23 ^a^	0.69 ± 0.29 ^a^	0.81 ± 0.04 ^b^
C18:1 trans 9 (Elaidic)	9.68 ± 0.54 ^d^	8.81 ± 0.76 ^c^	6.31 ± 0.47 ^a^	7.14 ± 0.81 ^b^	8.40 ± 2.09 ^c^	ND	6.86 ± 0.88 ^a^	7.75 ± 1.57 ^b^	8.20 ± 2.09 ^c^	9.41 ± 1.79 ^d^
C18:1 cis 9 (Oleic)	3.03 ± 0.36 ^c^	ND	1.9 ± 0.09 ^a^	2.05 ± 0.24 ^ab^	2.28 ± 0.27 ^b^	2.25 ± 0.06 ^b^	1.98 ± 0.22 ^a^	2.96 ± 0.75 ^a^	2.11 ± 0.79 ^ab^	2.45 ± 0.07 ^b^
C20:1 cis 11 (Eicosenoic)	ND	ND	ND	ND	ND	0.59 ± 0.00 ^b^	0.51 ± 0.00 ^a^	0.62 ± 0.00 ^b^	0.70 ± 0.00 ^c^	0.68 ± 0.19 ^c^
C22:1 cis 13 (Erucanoic)	ND	3.64 ± 0.59 ^b^	2.54 ± 0.00 ^a^	2.63 ± 0.16 ^a^	ND	ND	ND	ND	ND	ND
C24:1 cis 15 (Nervonic)	ND	6.13 ± 0.48	ND	ND	ND	ND	ND	ND	ND	ND
C18:2 cis 9,12 (Linoleic)	11.73 ± 0.68 ^d^	ND	ND	ND	10.51 ± 2.70 ^c^	9.69 ± 0.23 ^b^	8.22 ± 0.83 ^a^	10.63 ± 1.79 ^b^	9.64 ± 1.86 ^b^	11.18 ± 2.20 ^d^
C18:3 cis 6,9,12 gamma (gamma-Linolenic)	ND	ND	ND	1.24 ± 0.08 ^b^	1.10 ± 0.00 ^b^	ND	ND	0.70 ± 0.00 ^a^	ND	ND
C18:3 cis 9,12,15 alpha (alpha-Linolenic)	ND	ND	ND	ND	1.15 ± 0.28	ND	ND	ND	0.66 ± 0.00	ND
C20:2 cis 11,14 (Eicosadienoic)	1.18 ± 0.05 ^b^	ND	0.81 ± 0.08 ^a^	0.90 ± 0.11 ^ab^	1.04 ± 0.33	0.90 ± 0.03 ^ab^	0.78 ± 0.08 ^a^	1.98 ± 0.13 ^ab^	ND	1.14 ± 0.33 ^b^
C20:4 cis 5,8,11,14 (Eicosatetraenoic)	4.00 ± 0.24 ^c^	ND	2.62 ± 0.20 ^a^	2.99 ± 0.31 ^b^	3.15 ± 0.25 ^b^	3.16 ± 0.07 ^b^	2.67 ± 0.23 ^a^	3.75 ± 0.96 ^a^	1.98 ± 1.35 ^a^	3.16 ± 0.15 ^b^
C20:5 cis 5,8,11,14,17 EPA (Eicosapentaenoic)	6.84 ± 0.38 ^b^	ND	4.73 ± 0.38 ^a^	5.18 ± 0.62 ^ab^	6.44 ± 1.59 ^b^	5.70 ± 0.18 ^b^	4.83 ± 0.44 ^a^	6.03 ± 0.85 ^b^	5.83 ± 0.83 ^b^	6.75 ± 1.67 ^b^
C22:6 cis 4,710,13,16,19 DHA (Docosahexaenoic)	8.41 ± 0.54 ^d^	8.00 ± 0.96 ^d^	5.82 ± 0.53 ^a^	6.37 ± 0.77 ^b^	7.25 ± 0.77 ^c^	6.82 ± 0.33 ^b^	5.96 ± 0.56 ^a^	7.39 ± 2.10 ^b^	6.30 ± 1.65 ^b^	6.49 ± 0.51 ^c^
Saturated fatty acid	44.47 ± 9.37	69.59 ± 7.70	67.98 ± 6.30	60.25 ± 9.16	40.45 ± 2.6	61.51 ± 2.75	48.75 ± 6.20	37.28 ± 17.02	52.78 ± 5.91	48.30 ± 5.76
Monounsaturated fatty acid	31.59 ± 2.00	31.51 ± 1.83	24.44 ± 1.27	24.65 ± 2.89	25.24 ± 2.0	17.34 ± 0.52	21.60 ± 2.45	28.70 ± 6.71	23.78 ± 7.57	23.78 ± 7.17
Polyunsaturated fatty acid	32.16 ± 1.89	8.00 ± 0.96	13.98 ± 1.20	16.68 ± 1.81	30.64 ± 5.94	26.27 ± 0.84	22.46 ± 2.15	30.48 ± 5.84	24.41 ± 6.08	28.72 ± 5.70

Values are presented as the mean ± standard deviation (*n* = 3). Different lowercase letters within the same row indicate significant differences (*p* ˂ 0.05). ND: not detected.

**Table 2:** Likeness score of Pacific white shrimp treated without and with the PEF-1% SLE-VI process and MAP and without and with CP treatment on day 0 and the selected sample stored at 4 °C for 18 days.

**Storage Time (Days)**	**Sample**	**Appearance**	**Color**	**Texture**	**Taste**	**Flavor**	**Odor**	**Overall**
0	Control	7.90 ± 0.88 ^a^	8.00 ± 0.67 ^a^	8.10 ± 0.57 ^a^	8.00 ± 0.67 ^a^	8.10 ± 0.57 ^a^	8.00 ± 0.82 ^a^	8.10 ± 0.57 ^a^
	CP	8.09 ± 0.83 ^a^	8.00 ± 0.77 ^a^	8.36 ± 0.67 ^a^	7.91 ± 0.83 ^a^	8.18 ± 0.60 ^a^	7.91 ± 0.54 ^a^	8.27 ± 0.65 ^a^
	PEF-SLE1-VI-MAP1	8.00 ± 0.67 ^a^	8.10 ± 1.10 ^a^	7.80 ± 0.63 ^a^	7.80 ± 0.63 ^a^	8.10 ± 0.74 ^a^	8.00 ± 0.67 ^a^	8.20 ± 0.63 ^a^
	PEF-SLE1-VI-MAP2	7.90 ± 0.57 ^a^	7.80 ± 0.79 ^a^	7.90 ± 0.57 ^a^	8.10 ± 0.57 ^a^	7.90 ± 0.57 ^a^	7.90 ± 0.32 ^a^	8.00 ± 0.47 ^a^
	PEF-SLE1-VI-MAP3	7.90 ± 0.99 ^a^	8.00 ± 0.82 ^a^	7.90 ± 0.74 ^a^	7.70 ± 0.82 ^a^	8.00 ± 0.67 ^a^	7.90 ± 0.74 ^a^	7.90 ± 0.57 ^a^
	PEF-SLE1-VI-MAP1-CP	8.10 ± 0.74 ^a^	8.10 ± 0.88 ^a^	7.90 ± 0.57 ^a^	8.40 ± 0.52 ^a^	8.10 ± 0.74 ^a^	8.40 ± 0.52 ^a^	8.30 ± 0.48 ^a^
	PEF-SLE1-VI-MAP2-CP	8.30 ± 0.67 ^a^	7.90 ± 0.74 ^a^	8.10 ± 0.88 ^a^	8.20 ± 0.79 ^a^	8.00 ± 0.82 ^a^	8.20 ± 0.63 ^a^	8.10 ± 0.57 ^a^
	PEF-SLE1-VI-MAP3-CP	8.1 ± 0.88 ^a^	8.30 ± 0.67 ^a^	8.10 ± 0.88	7.90 ± 0.88 ^a^	8.00 ± 0.67 ^a^	8.10 ± 0.57 ^a^	8.20 ± 0.63 ^a^
18	PEF-SLE1-VI-MAP3-CP	6.30 ± 1.06 ^b^	6.40 ± 0.84 ^b^	6.40 ± 1.07 ^b^	6.60 ± 1.07 ^b^	6.20 ± 0.92 ^b^	6.30 ± 1.06 ^b^	6.40 ± 0.84 ^b^

Values are presented as the mean ± standard deviation (*n* = 50). Different lowercase letters within the same column indicate significant differences (*p* ˂ 0.05).

## Data Availability

The original contributions presented in this study are included in the article. Further inquiries can be directed to the corresponding author.
